# Plasma cell-free DNA (cfDNA) as a predictive and prognostic marker in patients with metastatic breast cancer

**DOI:** 10.1186/s13058-019-1235-8

**Published:** 2019-12-19

**Authors:** Daniel Fernandez-Garcia, Allison Hills, Karen Page, Robert K. Hastings, Bradley Toghill, Kate S. Goddard, Charlotte Ion, Olivia Ogle, Anna Rita Boydell, Kelly Gleason, Mark Rutherford, Adrian Lim, David S. Guttery, R. Charles Coombes, Jacqueline A. Shaw

**Affiliations:** 1 Leicester Cancer Research Centre, Department of Genetics and Genome Biology, University of Leicester, Robert Kilpatrick Clinical Sciences Building, Leicester Royal Infirmary, Leicester, LE2 7LX UK; 2 Department of Surgery and Cancer, Imperial College London, Du Cane Road, Hammersmith, London, W12 0NN UK; 3 Imperial College London and Healthcare NHS Trust, Charing Cross Hospital, Fulham Palace Road, London, W6 8RF UK; 4 Welsh School of Pharmacy and Pharmaceutical Sciences, Redwood Building, King Edward VII Ave, Cardiff, CF10 3NB UK; 5 Department of Health Sciences, University of Leicester, George Davies Centre, Leicester, LE1 7RH UK

**Keywords:** Breast cancer, Liquid biopsy, Biomarkers, cfDNA, CTCs

## Abstract

**Background:**

Breast cancer (BC) is the most common cancer in women, and despite the introduction of new screening programmes, therapies and monitoring technologies, there is still a need to develop more useful tests for monitoring treatment response and to inform clinical decision making.

The purpose of this study was to compare circulating cell-free DNA (cfDNA) and circulating tumour cells (CTCs) with conventional breast cancer blood biomarkers (CA15-3 and alkaline phosphatase (AP)) as predictors of response to treatment and prognosis in patients with metastatic breast cancer (MBC).

**Methods:**

One hundred ninety-four female patients with radiologically confirmed MBC were recruited to the study. Total cfDNA levels were determined by qPCR and compared with CELLSEARCH® CTC counts and CA15-3 and alkaline phosphatase (AP) values. Blood biomarker data were compared with conventional tumour markers, treatment(s) and response as assessed by RECIST and survival.

Non-parametric statistical hypothesis tests were used to examine differences, correlation analysis and linear regression to determine correlation and to describe its effects, logistic regression and receiver operating characteristic curve (ROC curve) to estimate the strength of the relationship between biomarkers and clinical outcomes and value normalization against standard deviation to make biomarker values comparable. Kaplan–Meier estimator and Cox regression models were used to assess survival. Univariate and multivariate models were performed where appropriate.

**Results:**

Multivariate analysis showed that both the amount of total cfDNA (*p* value = 0.024, HR = 1.199, CI = 1.024–1.405) and the number of CTCs (*p* value = 0.001, HR = 1.243, CI = 1.088–1.421) are predictors of overall survival (OS), whereas total cfDNA levels is the sole predictor for progression-free survival (PFS) (*p* value = 0.042, HR = 1.193, CI = 1.007–1.415) and disease response when comparing response to non-response to treatment (HR = 15.917, HR = 12.481 for univariate and multivariate analysis, respectively). Lastly, combined analysis of CTCs and cfDNA is more informative than the combination of two conventional biomarkers (CA15-3 and AP) for prediction of OS.

**Conclusion:**

Measurement of total cfDNA levels, which is a simpler and less expensive biomarker than CTC counts, is associated with PFS, OS and response in MBC, suggesting potential clinical application of a cheap and simple blood-based test.

## Introduction

Breast cancer (BC) is the most common cancer in women [1]. The introduction of screening programmes and the development of targeted therapies has significantly improved BC survival rates in the last 40 years [2, 3]. However, although many patients are initially responsive to therapies, resistance can develop and lead to relapse and ultimately death from metastatic disease [4]. Patients with metastatic breast cancer (MBC) are monitored by radiological imaging; primarily by computed tomography (CT) on average every 3 months, and scans are assessed using Response Evaluation Criteria in Solid Tumour (RECIST) criteria to determine disease response to treatment. FLT-PET and magnetic resonance imaging (MRI) is also carried out in some centres, but these tests are costly, insensitive and less readily accessible. Alongside imaging, cancer antigen 15-3 (CA15-3) and alkaline phosphatase (AP) are often measured, although these lack in both sensitivity and specificity [5, 6]. Therefore, there is a need to develop more useful tests for monitoring treatment response and to inform clinical decision making.

Blood-based biomarkers, including circulating cell-free DNA (cfDNA) and circulating tumour cells (CTCs) have attracted considerable attention in recent years due to their potential as minimally invasive tools for cancer monitoring. A CTC count of ≥ 5 CTCs/7.5 ml blood, as determined by CellSearch®, is an independent predictor of poor prognosis in MBC, irrespective of other clinical parameters [7]. CTC counts can also be used to guide therapy selection in newly diagnosed patients receiving first-line systemic treatment [7, 8]. Previous studies have proposed that CTC counts are superior to conventional radiological measures as predictor of prognosis and response in patients with MBC [8, 9], but as yet, the application of total cfDNA levels has not been widely studied in MBC [10, 11]. Circulating cfDNA is derived from a combination of apoptosis, necrosis and active secretion from cancer cells and is found at higher levels in patients with advanced cancer than in either healthy individuals [12] or patients with early-stage disease [13]. The tumour-derived fraction of this total cfDNA, termed circulating tumour DNA (ctDNA), is under wide investigation as a prognostic biomarker in several types of cancer, including breast, lung and colon cancers [14–17]. Although much ongoing research is focussed on profiling of ctDNA, these analyses are currently expensive and not yet established in the clinic. We therefore compared conventional breast cancer blood biomarkers (CA15-3 and alkaline phosphatase (AP)) with CTC counts and simple measurement of total cfDNA levels, rather than ctDNA profiles to assess the best predictor of response to treatment and prognosis in 194 patients with metastatic breast cancer. The results indicate that measurement of total cfDNA levels is a good predictor of response and survival in patients with MBC suggesting potential clinical application of a cheap and simple blood-based test.

## Methods

### Patients and demographics

Between February 2012 and July 2016, 194 female patients with radiologically confirmed MBC, attending the breast oncology clinic at Charing Cross Hospital, London, were recruited to this study. One hundred ninety-three patients had proven metastatic disease, and one had unresectable locally recurrent disease.

Thirty-two patients of the 194 patients were off treatment at the time of blood sampling, and the remainder was receiving treatment for MBC. Details of treatment(s) undergone by each patient at the time of blood sample collection were obtained from the Imperial College NHS electronic prescription system and where necessary, patient records. CT and MRI data were obtained from patient records and results confirmed by a consultant radiologist to determine patient disease status at time of blood collection. There were three orthogonal measurements of the primary tumour and volume estimated using a volume calculator, as described previously [18].

The maximal dimensions of the largest metastases were provided—two max per organ as per RECIST criteria. If there were multiple widespread lesions, then an estimate of how much of the whole organ is infiltrated with tumour was estimated visually. These were typically lymph nodes, lungs, liver and occasional brain and adrenal metastasis.

Only lytic bony lesions were counted. Pleural and peritoneal diffuse disease was documented but only measured when there was a sizable mass. Irradiated bony or CNS sites were followed where hopefully there was a baseline but the disease may not be measurable.

Data from scans undergone at a time-point, generally within 2 weeks, closest to that of sample collection were used to evaluate disease response. Response to treatment was assessed using RECIST criteria [19]. A total of 30 patients were responding to their treatment, 73 had stable disease and 91 were progressing. The ER, PR and HER2 status of the primary tumour and metastatic biopsy where available were obtained from histology reports (Table 1; Additional file 1).

**Table 1:** Descriptive statistics of variables and clinical characteristics of patients included in the study

Clinicopathological feature, observed at time of sample			
Collection	Details	*n*	% of total
Age at time of sample collection (y*)	Median	59.50	–
	IQR*	20	–
	Range	29-89	–
Time spent in study^a^ (m*)	Median	23	–
	IQR*	27	–
	Range	1-59	–
Status^b^	Alive	101	52.1
	Deceased	93	47.9
ER status	Positive	157	80.9
	Negative	30	15.5
	Unknown	7	3.6
PR status	Positive	115	59.3
	Negative	55	28.4
	Unknown	24	12.4
HER2 status	Positive	35	18.0
	Negative	133	68.6
	Unknown	26	13.4
CTCs/7.5 ml blood, by CellSearch®	Median	0.000	–
	IQR*	4.000	–
	Range	0-6848	–
	0–4	148	76.3
	≥ 5	46	23.7
cfDNA yield (ng/μl)	Median	0.120	–
	IQR*	0.175	–
	Range	0.003-5.460	–
	< 0.306	146	75.3
	≥ 0.306	48	24.7
CA15-3 (U/ml)	Median	49.000	–
	IQR*	149.250	–
	Range	1-6313	–
	< 32	67	34.5
	≥ 32	127	65.5
AP (IU/L)	Median	89.500	–
	IQR*	46.750	–
	Range	30-555	–
	< 130	154	79.4
	≥ 130	40	20.6
Treatment patient undergoing at the time of blood sampling	Endocrine	95	49.0
	Chemotherapy^c^	49	25.3
	HER2-targeted therapy^d^	18	9.3
	Off treatment	32	16.5
Disease response at time of blood sampling	Responding	30	15.5
	Stable disease	73	37.6
	Progressive disease	91	46.9

After screening and recruitment, patients were followed-up through the study. HER2 status was determined by immunohistochemical and fluorescence in situ hybridization assays. A patient was considered to have HER2-positive cancer if either assay was positive. CA15-3 and AP levels were determined form patient notes

*IQR* interquartile range, *y* years, *m* months

^a^Time from collection to end of study or death

^b^Status of patients at the end of the study (deceased or alive)

^c^Chemotherapy +/− endocrine therapy

^d^HER2 therapy +/− endocrine therapy

Initially, a small subset of the population of only 36 breast cancer metastatic patients was selected to conduct pilot studies. The main aim was to obtain preliminary results regarding the effects of biomarkers on the different clinical variables included in the study, as well as to interrogate the possible implications of the tumour bulk, as tumour volume and number of metastatic sites, with the biomarkers or the clinical parameters included, as response or survival. This small subset of the population was used as well to determine the necessary sample size to carry out the study (Additional file 2).

### Measurement of biomarkers

**Fig. 1:**
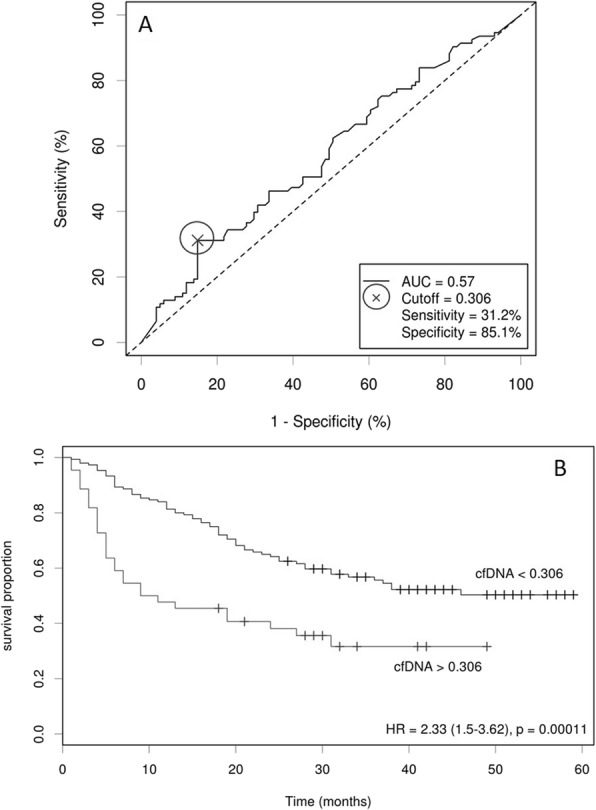
Determination of the optimum cut-off point for cfDNA yield according to the patient status (alive vs deceased). **A** ROC curves determine the cut-off point by minimizing the distance on the ROC curve to the left top edge of the diagram (100% sensitivity and 100% specificity), by minimizing the Manhattan distance between the points and the Youden’s index, which equals to maximize sensitivity and specificity. **B** Survival analysis defines the optimal cut-off as the point with the most significant (log-rank test) split. Hazard ratios (HRs) were calculated, including 95% confidence intervals. Cox regression and Kaplan–Meier analysis

Twenty milligrams of venous blood samples were collected into K_2_EDTA tubes and processed by double centrifugation to obtain plasma. Total cfDNA was extracted from 3 ml plasma using the Circulating Nucleic Acids kit (Qiagen) and quantified through a StepOnePlus Real-Time PCR System (Applied Biosystems) using a 96 bp single copy TaqMan assay as described previously [20]. 7.5 ml blood was separately collected into a CellSave Preservative tube and processed and counted within 96 h of collection using the CELLSEARCH® Circulating Tumor Cell Kit (Menarini Silicon Biosystems), as described previously [21, 22]. A threshold of 5 EpCAM^+^ CTC per 7.5 ml blood was selected to categorize the biomarker to low count (< 5 CTCs) and high counts (≥ 5 CTCs), based on previous studies [7, 23]. The total cfDNA yield was categorized by optimizing the correlation with clinical outcome based on ROC curve analysis [10, 24, 25] and by analysing the significance of the correlation with survival [24]. Both methods showed a threshold of 0.306 ng/μl cfDNA above which levels were considered elevated (Fig. 1).

CA15-3 and alkaline phosphatase (AP) values were obtained from patient records. The upper limits of normal values were 32 U/ml for CA15-3 and 130 IU/ml for AP, respectively, in accordance with the clinical reference ranges used routinely at Charing Cross Hospital.

### Statistical analysis

Mann-Whitney-Wilcoxon and Kruskal-Wallis non-parametric tests were used, when appropriate, to examine differences between baseline characteristics of the patients. Spearman’s rank correlation coefficient and linear regression with Fishers test *p* value were used to determine whether the variables were correlated or not, and logistic regression with Wald statistic *p* value and receiver operating characteristic curve (ROC curve) with the area under the curve (AUC) were used to estimate the strength of the relationship between biomarkers and clinical outcomes. To make each of the 4 biomarkers comparable as a continuous variable, each biomarker was normalised against its own standard deviation. Univariate and multivariate models were performed where appropriate. Kaplan–Meier estimator and Cox regression models were used to assess survival (overall survival (OS) and progression-free survival (PFS)). Each model was constructed using the counting process notation (start, end, event) [26], where the date of blood collection was taken as the start and the date of last follow-up, date of progression or date of death was considered the end, with an agreed administrative censoring date of 31 July 2017. Survival curves were compared using the log-rank test. Cox proportional-hazards regression analysis was used to estimate univariate and multivariate hazard ratios for progression-free survival and overall survival. All statistical analyses were performed using SPSS 25.0 software. Power and sample size analysis were performed using R package “pwr” version 1.2-2 (R version 3.5.1).

## Results

### Patient characteristics, treatment and disease response

An initial analysis was carried out using a subset of 36 patients to power the minimum number of patient samples needed for the study. To perform the analysis, we used the presence/absence of CTC counts against the status (alive/deceased) of 36 patients included in this preliminary study for whom tumour volume data was available. The effect size was calculated according to the means of the population and the pooled standard deviation of those means, which is the square root of the average of the two standard deviations. A total of 20 patients (10 patients in each group) are needed to achieve 80% power at two-sided 5% significance level; however, in order to maximize the effect and precision of the results of our study and in order to capture the full interaction between all the biomarkers and clinical parameters included, we opted to analyse all patients recruited over a fixed time period, between February 2012 and July 2016, which increased the number of patients to 194.

In this pilot study over the subset of 36 patients, there was no association of the biomarkers with tumour bulk, either by the total tumour volume or the number of metastatic sites. Logistic regression analysis showed that none of the biomarkers, as categorical variables or as continuous variables using a univariate or multivariate approach, nor response by RECIST criteria was associated to tumour bulk (Additional file 3).

Concerning the total cohort of 194 patients, the median patient age was 59.5 years (ranges from 29 to 89; IQR = 20) and patients were from a range of breast cancer sub-types as determined by ER, PR and HER2 status. Of the whole cohort, 95 (49.0%) patients were undergoing endocrine treatment, 49 (25.3%) were receiving on chemotherapy, 18 (9.3%) were on HER2-targeted therapy, and 32 (16.5%) were off treatment at the time of blood sample (Table 1). Disease response data was obtained for all 194 patients using RECIST criteria [27] (Table 1; Additional file 4). There were 30 (15.5%) patients showing a complete or partial response, 73 (37.6%) patients with stable disease and 91 patients (46.9%) with progressive disease.

### Blood biomarkers

Serum CA15-3 levels were elevated (≥ 32 U/ml) in 127 patients (65.5%) (70 [55.1%] progressing, 34 [26.8%] with a stable disease and 23 [18.1%] responding), AP was elevated (≥ 130 IU/L) in 40 patients (20.6%) (31 progressing [77.5%], 7 [17.5%] with a stable disease and 2 [5.0%] responding) and both markers were elevated in 38 (19.6%) patients (29 progressing [76.3%], 7 [18.4%] with a stable disease and 2 [5.3%] responding) (Additional file 4) at the time of blood sampling. Levels of CA15-3 and AP were significantly correlated (*p* value < 0.0001; Table 2), confirming previous reports [6, 21, 28]. This correlation was confirmed by linear regression analysis (*p* value < 0.0001; Additional file 5) and logistic regression analysis, where CA15-3 values were associated with AP values, and vice versa (*p* value < 0.0001, OR = 13.876, CI = 3.231–59.597; Additional files 6 and 7).

**Table 2:** Non-parametric correlation analysis of the four biomarkers included in the study

Non-parametric correlations (Spearman’s rho)				
	CTC count	cfDNA yield	CA15-3	ALP
CTC count				
Correlation coefficient				
*p* value				
cfDNA yield				
Correlation coefficient	0.294			
*p* value	*< 0.0001*			
CA15-3				
Correlation coefficient	0.464	0.247		
*p* value	*< 0.0001*	*0.001*		
AP				
Correlation coefficient	0.392	0.193	0.370	
*p* value	*< 0.0001*	*0.007*	*< 0.0001*	

Eighty-nine of 194 patients (45.9%) had at least one EpCAM^+^-positive CTC per 7.5 ml blood analysed (range 1–6848) and 46 patients (23.7%) had ≥ 5 CTCs per 7.5 ml blood (defined as a high CTC count) [7] (33 progressing [71.7%], 11 [23.9%] with a stable disease and 2 [4.4%] responding). The median plasma cfDNA concentration was 0.112 ng/μl (IQR = 0.095; range 0.003 to 5.46 ng/μl), and 44 patients (22.7%) had high levels of cfDNA in plasma (≥ 0.306 ng/μ) (33 progressing [75.0%], 11 [25.0%] with stable disease). None of the patients with high cfDNA levels were responding to treatment at the time of blood sampling (Table 1; Additional file 4). The number of CTCs detected correlated positively with the total cfDNA level (*p* value < 0.0001), as reported previously [21] (Table 2). This correlation was confirmed by logistic regression analysis, where high CTC counts were associated with cfDNA overall levels (*p* value = 0.003, OR = 2.140, CI = 1.305-3.510 and *p* value = 0.007, OR = 2.028, CI = 1.212–3.394; univariate and multivariate analysis; Additional file 6) and where high CTC counts were associated with high cfDNA levels, and vice versa (*p* value < 0.0001, OR = 8.083, CI = 3.803–17.180; Table 3). All 4 blood biomarkers included in the study were significantly correlated (Table 2), confirmed by regression analysis (Additional files 6 and 7).

**Table 3:** Logistic regression analysis between stratified clinical parameter (response to treatment) and stratified biomarkers, showing the *p* value, odds ratio and the 95% confidence interval for this odds ratio

Categorical variable to analyse	Variable	*p* value	Odds ratio	95% CI for OR		Type of analysis	Type of variable
				Lower	Upper		
Response (RECIST 3 categories) vs							
Responding	CTC threshold	Reference category				Univariate	Stratified biomarker
Stable disease		0.256	2.484	0.516	11.955		
Progressive disease		*0.007*	*7.966*	1.783	35.587		
Responding	cfDNA threshold	*Cannot be quantified				Univariate	Stratified biomarker
Stable disease							
Progressive disease							
Responding	CA15-3 threshold	Reference category				Univariate	Stratified biomarker
Stable disease		*0.007*	*0.265*	0.101	0.695		
Progressive disease		0.977	1.014	0.382	2.694		
Responding	AP threshold	Reference category				Univariate	Stratified biomarker
Stable disease		0.635	1.485	0.29	7.597		
Progressive disease		*0.01*	*7.233*	1.616	32.373		

*Cannot be quantified as all patients in the group responding to treatment have low levels of cfDNA. No patients with high levels of cfDNA responding to treatment

### Comparison of biomarkers with molecular subtype and response to treatment

Based on data from the cohort, we also categorised each biomarker according to high and low cut-off points/thresholds [7, 14, 23–25]. We then compared the four biomarkers against various clinical parameters using both the threshold values and using each biomarker as a continuous variable (Additional files 8 and 9).

Regarding breast cancer molecular subtypes, analysis of each biomarker showed few significant results regarding hormone receptor (ER/PR) or HER2 status, or type of therapy administered. When using categorized variables, CTCs and CA15-3 higher values were associated with HER2-negative status (*p* value = 0.048, OR = 0.284, CI = 0.082–0.989; *p* value = 0.003, OR = 0.311, CI = 0.145–0.670) and higher CA15-3 was associated with patients receiving chemotherapy treatment. Results as continuous variables showed CA15-3 was associated with ER and PR status (*p* value = 0.036, OR = 16.416, CI = 1.204–223.876; *p* value = 0.015, OR = 5.511, CI = 1.394–21.792), as well as the use of chemotherapy treatment (*p* value = 0.027, OR = 1.495, CI = 1.046–2.136) by univariate and multivariate analysis. Lastly, levels of cfDNA and AP were associated with patients receiving HER2 therapy (*p* value = 0.028, OR = 2.024, CI = 1.079–3.794; *p* value = 0.048, OR = 1.696, CI = 1.006–2.860) by multivariate analysis (Additional files 8 and 9).

According to RECIST criteria, patient response to treatment was classified as either complete or partial response, stable disease or progressive disease. To analyse the relationship with all four biomarkers, we compared the different categories against each biomarker as categorical variables (Table 3). Results showed that high CTC counts and high AP levels are predictors of progressive disease (*p* value = 0.007, OR = 7.996, CI = 1.783–35.587 and *p* value = 0.01, OR = 7.233, CI = 1.616–32.373, respectively), while CA15-3 is only associated with patients with a stable disease (*p* value = 0.007, OR = 0.265, CI = 0.101–0.695). It was not possible to quantify the effect of low/high levels of cfDNA because none of the 44 patients with higher levels of cfDNA were responding to treatment as all had either stable or progressive disease. We therefore decided not to use this response stratification to draw any conclusions.

To quantify accurately the effect of all biomarkers as a response predictor, we opted to group the response into two categories only. For that task, there were two different approaches: group categories by disease status (progressing vs non-progressing disease) or focus the matter into the prediction of treatment response (responding vs non-responding to treatment). Based on clinical advice, we decided that assessing the efficiency of the treatment (responders vs non-responders) would be most useful in the follow-up of patients. Therefore, each biomarker was compared as a categorical and continuous variable against response to treatment (Table 4).

**Table 4:** Logistic regression analysis between stratified clinical parameter (response to treatment) and stratified or normalized continuous biomarkers, showing the *p* value, odds ratio and the 95% confidence interval for this odds ratio. Univariate and multivariate analysis

Categorical variable to analyse	Variable	*p* value	Odds ratio	95% CI for OR		Type of analysis	Type of variable
				Lower	Upper		
Response (RECIST 2 categories) vs	CTC threshold	*0.030*	*5.133*	1.174	22.450	Univariate	Stratified biomarker
	cfDNA threshold	*Cannot be quantified					
	CA15-3 threshold	0.165	0.528	0.214	1.302		
	AP threshold	0.056	4.222	0.961	18.542		
	CTC threshold	0.265	2.502	0.499	12.534	**Multivariate	
	cfDNA threshold	*Cannot be quantified					
	CA15-3 threshold	*0.012*	*0.297*	0.116	0.766		
	AP threshold	0.171	3.070	0.616	15.298		
Response (RECIST 2 categories) vs	CTC count	0.405	27.561	0.011	67106.275	Univariate	Normalized continuous biomarker
	cfDNA yield	*0.035*	*15.917*	1.223	207.106		
	CA15-3 levels	0.722	0.938	0.661	1.331		
	AP levels	0.139	1.611	0.856	3.031		
	CTC count	0.809	1.445	0.073	28.702	**Multivariate	
	cfDNA yield	*0.055*	*12.481*	0.946	164.576		
	CA15-3 levels	0.290	0.814	0.556	1.192		
	AP levels	0.212	1.575	0.771	3.215		

*Cannot be quantified as all patients in the group responding to treatment have low levels of cfDNA. No patients with high levels of cfDNA responding to treatment

**Multivariate analysis represent the combination and analysis of all biomarkers (CTC count, cfDNA yield, CA15-3 levels and AP levels) together, as a joint analysis, to explore the possible interactions between them and how this interactions affects their effect on the clinical variables under study

The analysis of the 4 biomarkers as continuous variables showed cfDNA levels as the sole predictor for treatment response, establishing a clear separation between responding and non-responding patients, either by univariate (*p* value = 0.035, OR = 15.917) or multivariate analysis (*p* value = 0.055*, OR = 12.481; *borderline value) (Table 4).

**Fig. 2:**
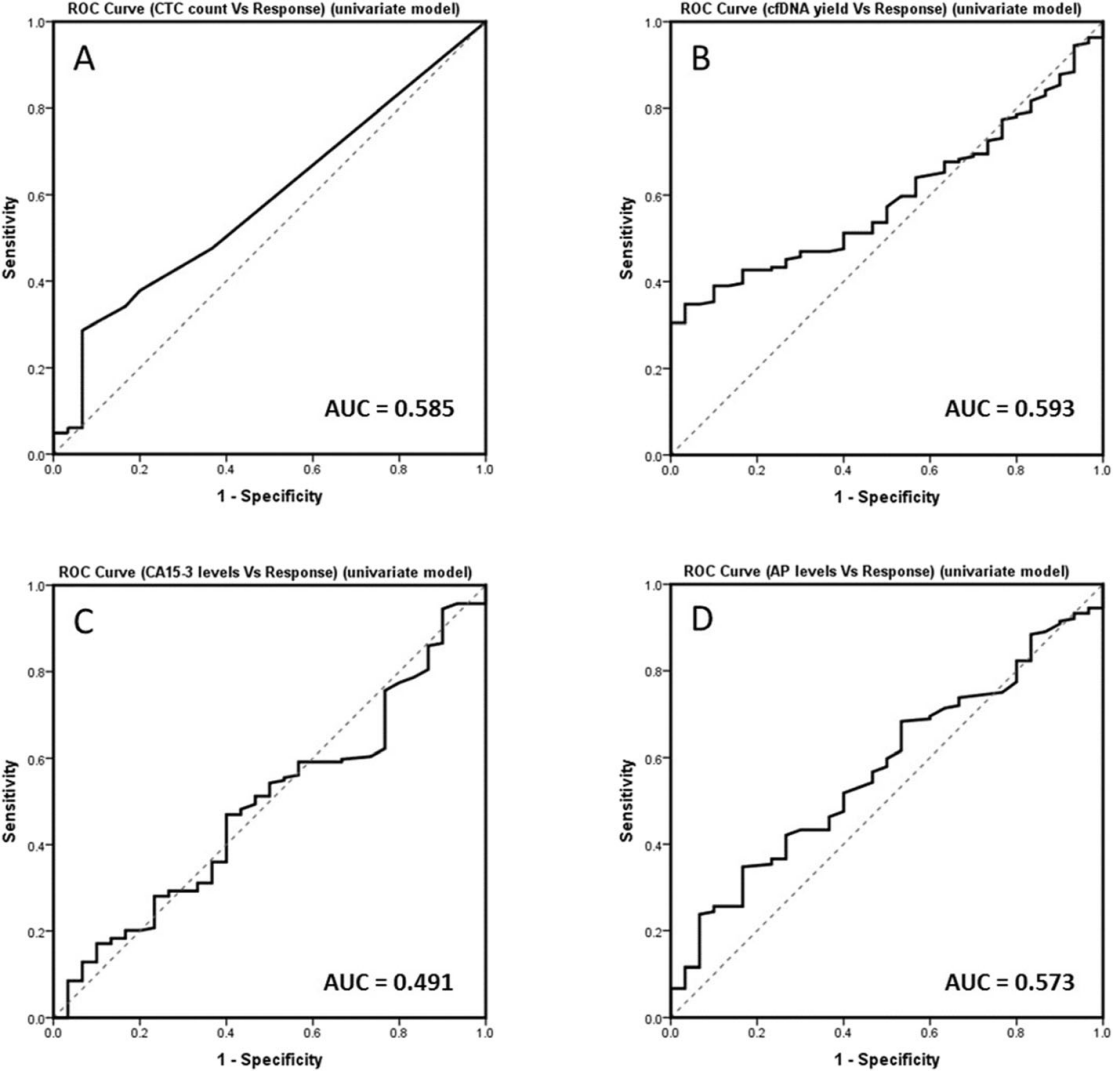
ROC curve analysis to determinate the power of discrimination of the biomarkers between different patient response. Univariate approach for (**A**) CTC counts (AUC = 0.585; sensitivity = 47.6%; specificity = 63.3%), **B** cfDNA yield (AUC = 0.593; sensitivity = 59.8%; specificity = 46.7%), **C** CA15-3 levels (AUC = 0.491; sensitivity = 54.9%; specificity = 46.7%) and **D** AP levels (AUC = 0.573, sensitivity = 68.3%, specificity = 46.7%)

ROC curve analysis (Fig. 2) showed similar results for cfDNA yield, CTC counts and AP levels (AUC of 0.593, 0.585 and 0.573, respectively), whereas CA15-3 had a lower AUC (0.491). This suggests that CA15-3 was the poorest biomarker to discriminate patients according to their response to treatment. Although the AUC is modest, the cfDNA discrimination power is still 10% higher than CA15-3, which is one of the currently used markers in routine clinical practice. Concerning sensitivity and specificity analysis, AP levels had the highest sensitivity (68.3%) followed by cfDNA (59.8%), CA15-3 (54.9%) and CTC (47.6%), while CTC counts showed highest specificity (63.3%) followed by cfDNA, CA15-3 and AP, all with the same value (46.7%).

We interrogated the possible relationship between biomarkers and the presence/absence of any line of treatment. Results were not significant for any of the biomarkers (data not shown).

### Comparison of biomarkers with patient survival

**Fig. 3:**
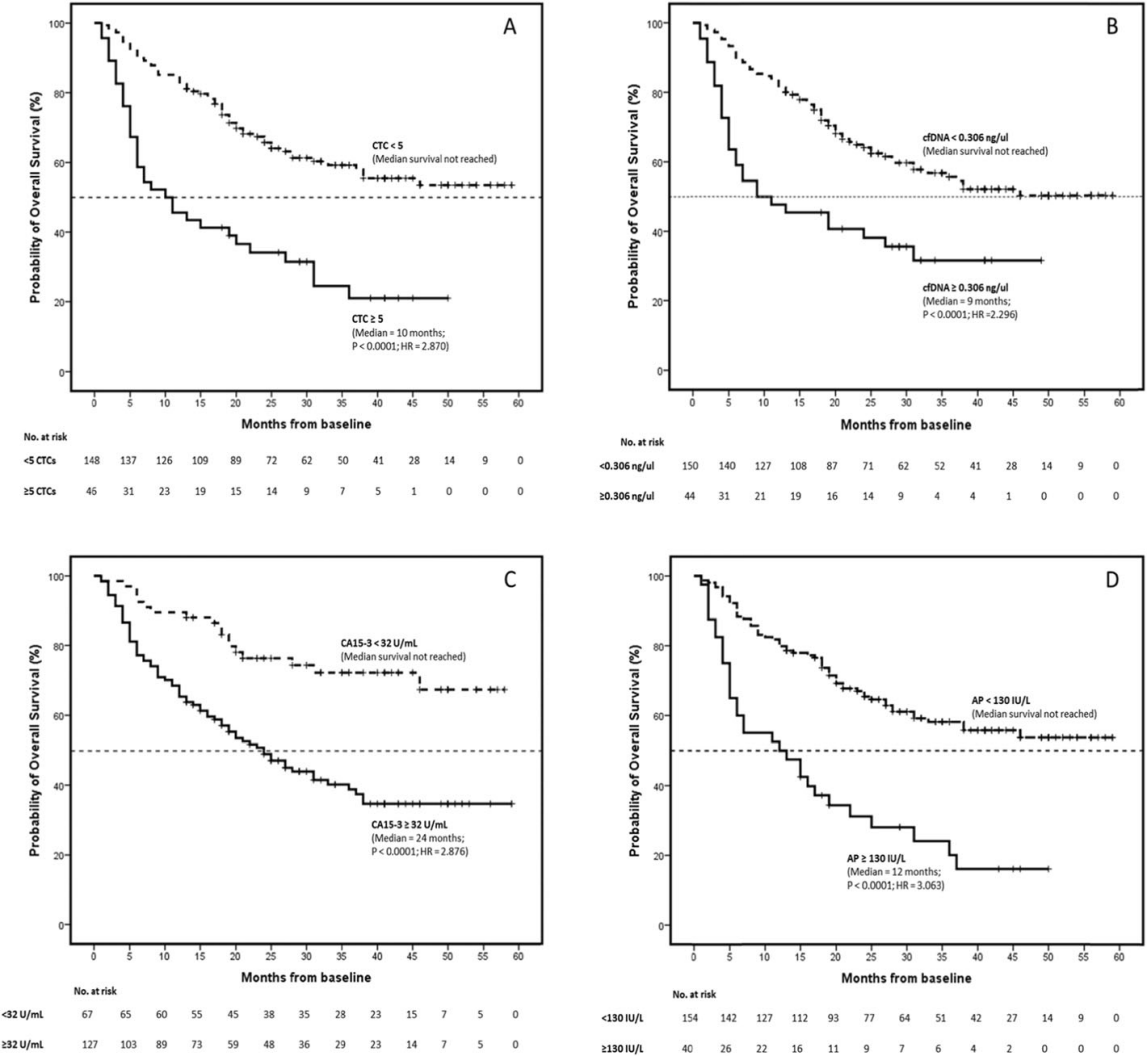
Kaplan–Meier estimates of probability of overall survival in patients with metastatic breast cancer. Comparison between patients with higher/lower **A** circulating tumour cells (*p* < 0.0001, chi-square = 26.372, hazard ratio = 2.870, 95% CI for HR = 1.876–4.392), **B** circulating free DNA (*p* < 0.0001, chi-square = 14.701; hazard ratio = 2.296, 95% CI for HR = 1.476–3.570), **C** CA15-3 (*p* < 0.0001, chi-square = 18.006; hazard ratio = 2.876, 95% CI for HR = 1.717–4.816) and **D** AP (*p* < 0.0001, chi-square = 28.710; hazard ratio = 3.063, 95% CI for HR = 1.982–4.732). *p* value obtained by the log-rank test. Hazard ratio and 95% CI obtained by Cox proportional-hazards regression analysis

Stratification of patients according to the different threshold values for each biomarker demonstrated, as expected, that higher counts/values of all the biomarkers were significantly associated with poorer overall survival (OS) (Fig. 3; Table 5). A strong relationship was observed between higher counts/levels of CTC (*p* value < 0.0001, HR = 2.870, CI = 1.876–4.392), cfDNA (*p* value < 0.0001, HR = 2.296, CI = 1.476–3.570), CA15-3 (*p* value < 0.0001, HR = 2.876, CI = 1.717–4.816) and AP (*p* value < 0.0001, HR = 3.063, CI = 1.982–4.732), with a poorer outcome of the metastatic breast cancer patients included in the study. However, when introducing the multivariate analysis approach, CTC fails as prognostic factors.

**Table 5:** Overall survival analysis of metastatic breast cancer patients, showing the *p* value, hazard ratio and the 95% confidence interval for this hazard ratio. Stratified or normalized continuous biomarkers used as variables. Univariate and multivariate analysis

Variable	*p* value	Hazard ratio	95% CI for HR		Type of analysis	Type of variable
			Lower	Upper		
Overall survival analysis						
CTC threshold	*< 0.0001*	*2.870*	1.876	4.392	Univariate	Stratified biomarker
cfDNA threshold	*< 0.0001*	*2.296*	1.476	3.570		
CA15-3 threshold	*< 0.0001*	*2.876*	1.717	4.816		
AP threshold	*< 0.0001*	*3.063*	1.982	4.732		
CTC threshold	0.162	1.447	0.862	2.430	*Multivariate	
cfDNA threshold	*0.034*	*1.684*	*1.041*	*2.726*		
CA15-3 threshold	*0.017*	*1.973*	*1.130*	*3.446*		
AP threshold	*0.006*	*1.996*	*1.219*	*3.268*		
CTC count	*0.003*	*1.213*	1.068	1.376	Univariate	Normalized continuous biomarker
cfDNA yield	*0.016*	*1.216*	1.037	1.427		
CA15-3 levels	*0.001*	*1.278*	1.110	1.472		
AP levels	*< 0.0001*	*1.611*	1.365	1.902		
CTC count	*0.001*	*1.243*	1.088	1.421	*Multivariate	
cfDNA yield	*0.024*	*1.199*	1.024	1.405		
CA15-3 levels	*0.168*	*1.120*	0.953	1.315		
AP levels	*< 0.0001*	*1.565*	1.307	1.874		

*Multivariate analysis represent the combination and analysis of all biomarkers (CTC count, cfDNA yield, CA15-3 levels and AP levels) together, as a joint analysis, to explore the possible interactions between them and how this interactions affects their effect on the clinical variables under study

**Fig. 4:**
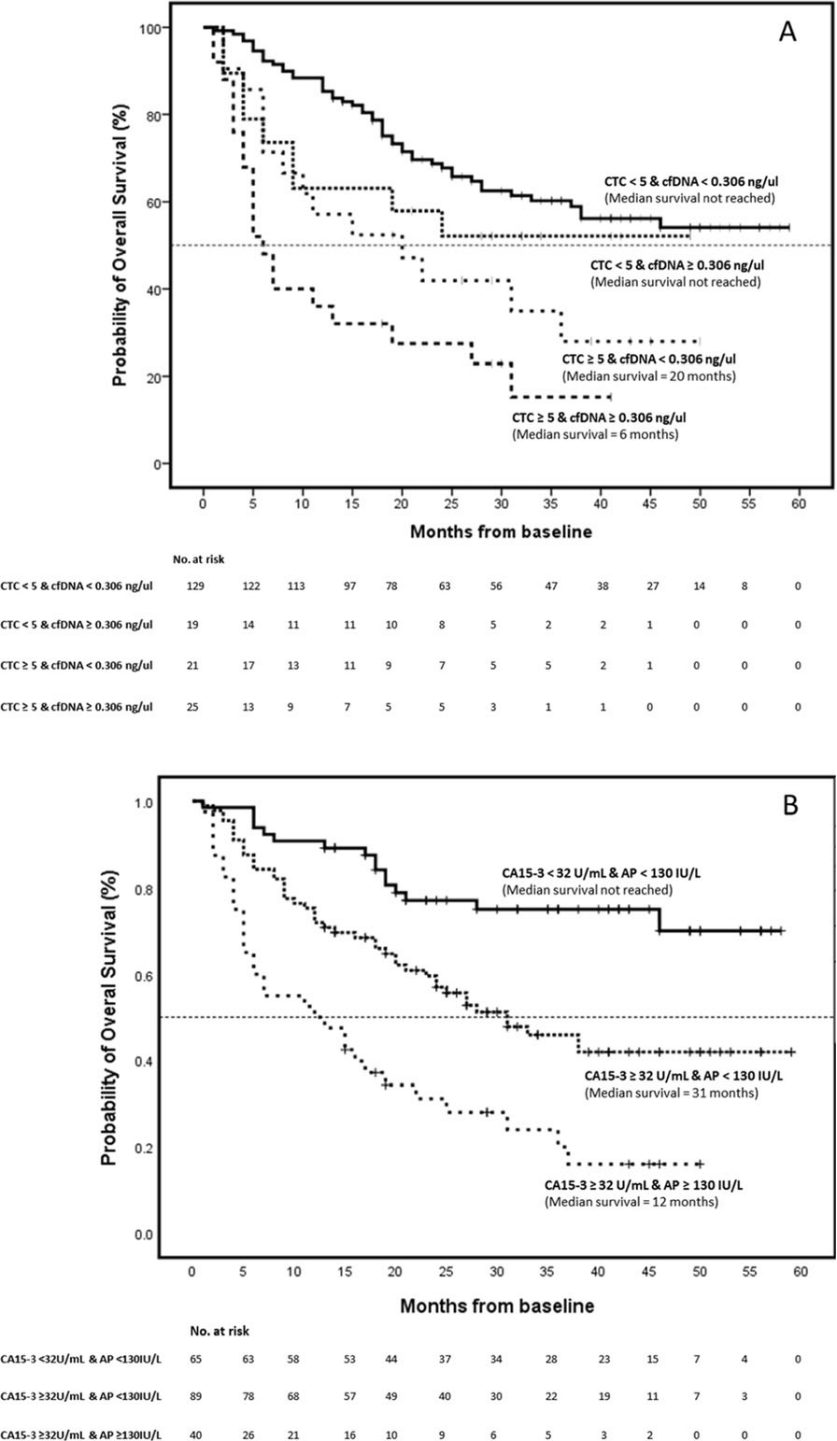
Kaplan–Meier estimates of probability of overall survival in patients with metastatic breast cancer. Joint analysis of **A** circulating tumour cells and circulating free DNA (*p* < 0.0001, chi-square = 33.718; hazard ratio for patients with ≥ 5 CTCs and < 0.306 ng/μl, 2.232 with 95% CI = 1.232–4.042; hazard ratio for patients with < 5 CTCs and ≥ 0.306 ng/μl, 1.479 with 95% CI = 0.726–3.010; hazard ratio for patients with ≥ 5 CTCs and ≥ 0.306 ng/μl, 4.047 with 95% CI = 2.394–6.841) and **B** levels of CA15-3 and AP (*p* < 0.0001; chi-square = 37.349; hazard ratio for patients with ≥ 32 U/mL and < 130 IU/L, 2.519 with 95% CI = 1.425–4.453; hazard ratio for patients with ≥ 32 U/mL and ≥ 130 IU/L, 5.568 with 95% CI = 3.033–10.223). Group with < 32 U/mL and ≥ 130 IU/L omitted in Fig. 5b because the low number of patients, and included into group with ≥ 32 U/mL and ≥ 130 IU/L. *p* value obtained by the log-rank test. Hazard ratio and 95% CI obtained by Cox proportional-hazards regression analysis

We also compared the combined effect of cfDNA levels and CTC counts and CA15-3 and AP levels, with OS (Fig. 4; Additional file 10). Overall survival was improved (median survival > 59 months) when the CTC counts were low (< 5 CTCs/7.5 ml blood) regardless of cfDNA levels. However, when CTC counts were high the median survival was significantly affected by cfDNA levels, being 20 months when cfDNA levels were low (*p* value = 0.008, HR = 2.232, CI = 1.232–4.042) and just 6 months when cfDNA levels were high (*p* value < 0.0001, HR = 4.047, CI = 2.394–6.841). High levels of CA15-3 also significantly affected the median overall survival, reducing the median survival time from > 59 months to 31 months when AP levels were low or 12 months when AP levels were high. However, combined analysis of CTC counts and cfDNA levels showed the largest effect on the overall survival of patients.

Using a Cox regression model to analyse all biomarkers as continuous variables, we observe that all four biomarkers were significantly associated with patients outcome by univariate analysis; however, when introducing multivariate analysis, only cfDNA levels (*p* value = 0.024, HR = 1.199, CI = 1.024–1.405), CTC counts (*p* value = 0.001, HR = 1.243, CI = 1.088–1.421) and AP levels (*p* value < 0.0001, HR = 1.565, CI = 1.307–1.874), but not CA15-3, were able to predict OS (Table 5).

**Fig. 5:**
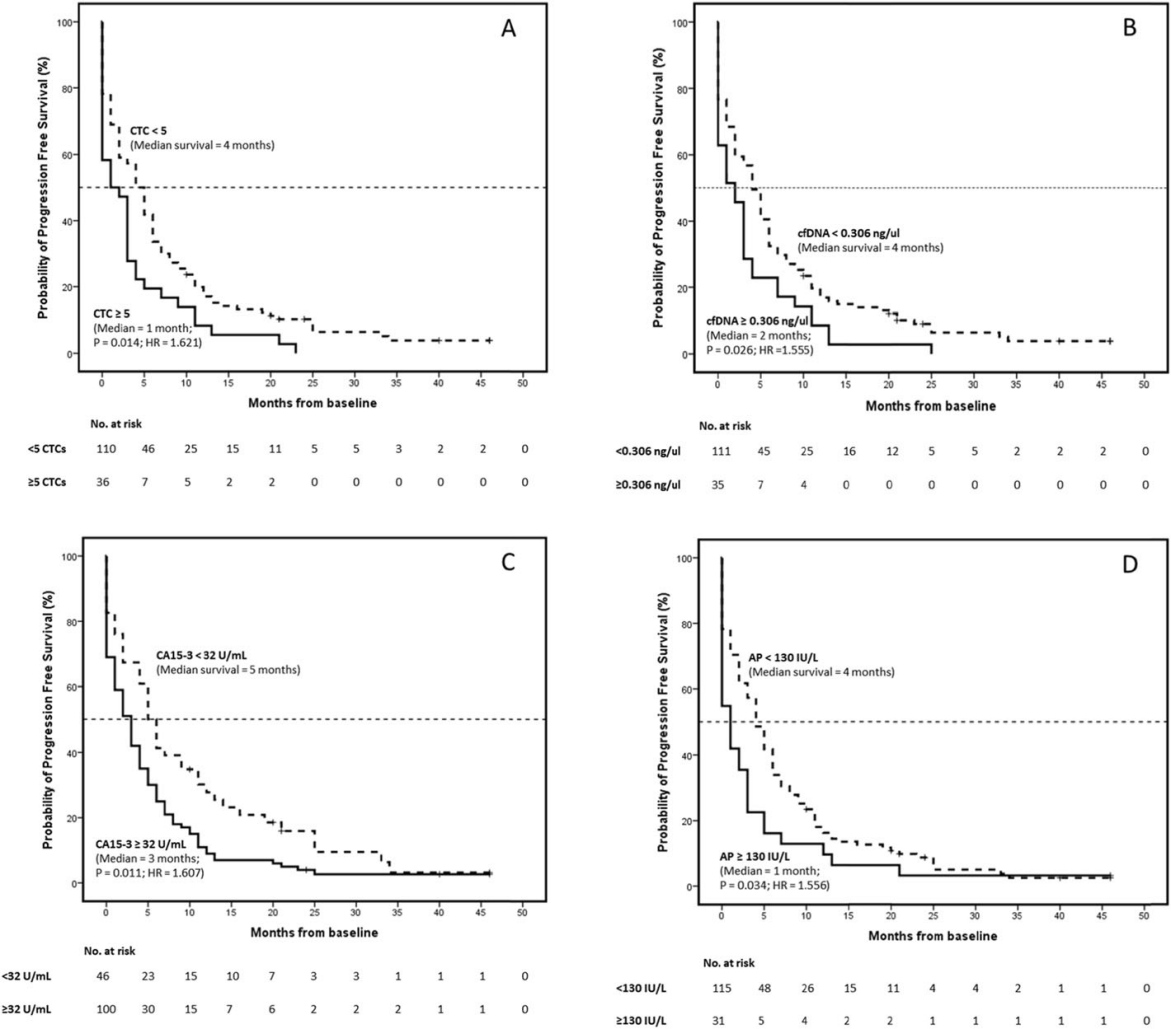
Kaplan–Meier estimates of probability of progression-free survival in patients with metastatic breast cancer. Comparison between patients with higher/lower **A** circulating tumour cells (*p* = 0.007; chi-square = 7.360; hazard ratio = 1.621; 95% CI for HR = 1.102-2.384), **B** circulating free DNA (*p* = 0.014; chi-square = 6.084; hazard ratio = 1.555; 95% CI for HR = 1.055–2.292), **C** CA15-3 (*p* = 0.006; chi-square = 7.689; hazard ratio = 1.607; 95% CI for HR = 1.113–2.319) and **D** AP (*p* = 0.019; chi-square = 5.494; hazard ratio = 1.556; 95% CI for HR = 1.034–2.340). *p* value obtained by the log-rank test. Hazard ratio and 95% CI obtained by Cox proportional-hazards regression analysis

Stratification of patients according to the threshold values also showed as expected that higher counts/values of all the biomarkers were significantly associated with poorer progression-free survival (PFS) by univariate analysis; however, none of them have significant results when using a multivariate approach (Fig. 5, Table 6). The analysis of all biomarkers as continuous variables showed that total cfDNA levels were the sole predictor of PFS by multivariate analysis (*p* value = 0.042, HR = 1.193, CI = 1.007–1.415) (Table 6). CTC counts and AP levels only had an effect as predictors in the univariate model, while CA15-3 levels did not have any effect.

**Table 6:** Progression-free survival analysis of metastatic breast cancer patients, showing the *p* value, hazard ratio and the 95% confidence interval for this hazard ratio. Stratified or normalized continuous biomarkers used as variables. Univariate and multivariate analysis

Variable	*p* value	Hazard ratio	95% CI for HR		Type of analysis	Type of variable
			Lower	Upper		
Progression-free survival analysis						
CTC threshold	*0.014*	*1.621*	1.102	2.384	Univariate	Stratified biomarker
cfDNA threshold	*0.026*	*1.555*	1.055	2.292		
CA15-3 threshold	*0.011*	*1.607*	1.113	2.319		
AP threshold	*0.034*	*1.556*	1.034	2.340		
CTC threshold	0.623	1.130	0.694	1.839	*Multivariate	
cfDNA threshold	0.171	0.731	0.467	1.145		
CA15-3 threshold	0.072	1.451	0.967	2.176		
AP threshold	0.461	1.187	0.753	1.872		
CTC count	*0.025*	*3.508*	1.172	10.499	Univariate	Normalized continuous biomarker
cfDNA yield	*0.013*	*1.233*	1.045	1.455		
CA15-3 levels	0.066	1.173	0.990	1.389		
AP levels	*0.037*	*1.220*	1.012	1.471		
CTC count	0.184	2.401	0.660	8.738	*Multivariate	
cfDNA yield	*0.042*	*1.193*	1.007	1.415		
CA15-3 levels	0.311	1.105	0.911	1.339		
AP levels	0.236	1.140	0.918	1.415		

*Multivariate analysis represent the combination and analysis of all biomarkers (CTC count, cfDNA yield, CA15-3 levels and AP levels) together, as a joint analysis, to explore the possible interactions between them and how this interactions affects their effect on the clinical variables under study

## Discussion

Many studies have shown that only approximately 50% of patients with MBC have high CTC counts or elevated CA15-3 [5, 6, 29]. Hence, many MBC patients do not have an acceptable blood marker that allows the clinician to monitor the outcome of therapy without recourse to expensive imaging. Further, exome sequencing of the tumour, although offering a personalised approach to mutation profiling through ctDNA [30], has limitations since progression after therapy is often followed by the emergence of clones expressing other mutations.

CA15-3 and AP are the two routine biomarkers currently used in the clinic. However, as neither appears to have a great ability to predict response to treatment, relapse or overall survival, in this study, we tried to compare these biomarkers with the newer biomarkers available (cfDNA and CTCs). Although the initial analysis of all four biomarkers showed that their ability to discriminate response to treatment is not great, based on sensitivity and specificity of each biomarker, an overall analysis of the area under the curve (AUC) by ROC curve analysis showed that cfDNA discriminatory power was 10% higher than CA15-3. This is, at least, a starting point to consider whether the markers we are using currently in the clinic are the best available.

The results of this study indicate that in patients with MBC, both the amount of total cfDNA and the number of EpCAM^+^ CTCs in patient blood during the treatment period, are reflective of disease response and are indicators of overall survival, and importantly, cfDNA levels are the best predictor of disease response and PFS. Meanwhile, the conventional biomarker CA15-3 failed as a predictor of response and survival (OS and PFS), when analysed as a continuous variable. Overall, both CTC counts and cfDNA levels were associated with clinical outcomes in patients with MBC both individually and jointly, providing independent validation to a recent study [10].

Whilst we have shown that higher CTC counts and cfDNA levels are individually predictive and prognostic in patients with MBC, analysis of both, as a paired test, provides additional prognostic information. Overall survival analysis in MBC patients based on CTC count alone showed a median survival > 59 months for patients with 0–4 CTCs/7.5 ml blood, compared with only 10 months for those with ≥ 5 CTCs/7.5 ml blood (Fig. 3). However, when patients have a CTC count ≥ 5 CTCs/7.5 ml blood, there was an increase in the median survival time when the patient’s levels of cfDNA are low (< 0.306 ng/μl) and a decrease when the levels of cfDNA are high (≥ 306 ng/μl) (Fig. 4). These results suggest that analysis of both CTC count and cfDNA level together may provide additional prognostic information and allow further stratification of patients with high CTC counts. One limitation of the current approach is the inability to detect CTCs in many patients even in the metastatic setting, and secondly that the majority of clinical service labs may not have access to a CTC platform, we therefore suggest that cfDNA measurement potentially represents an easier and less expensive biomarker than CTC counts in patients with MBC. Whilst our approach relies on detection of cfDNA levels rather than more specific mutation profiling through ctDNA, cfDNA measurement is a simple and inexpensive test that could be set up as a routine test and done as an adjunct to radiological assessment. Importantly, the cost per test is in the tens of dollars rather than the hundreds of dollars required currently for either CTC analysis or ctDNA profiling. Moreover, cfDNA measurement can be done more frequently than imaging through follow-up blood sampling to track disease response in real-time and alert clinicians as to when a change of treatment is needed. One other limitation to our study is that due to the high cost of CTC analysis, we analysed a single blood sample only from each patient; therefore, we cannot comment on dynamic responses over time.

Since we began this study in 2012, much research has focussed on characterising circulating tumour DNA (ctDNA), with the aim tracking somatic mutations with disease response. These studies have suggested that levels of ctDNA may be predictive of disease progression, overall survival and progression-free survival in different metastatic breast cancer patient populations, but these have not compared the results with other blood tests reflecting outcome. We explored the concordance between cfDNA and ctDNA levels in a previous study and showed that rising ctDNA is generally reflected by rising total cfDNA levels [19]. Some studies have reported that the dynamics of ctDNA show a better performance, when compared to the commonly assessed tumour protein biomarker CA15-3, in correlating with tumour burden, and provide a very early indication of treatment response [14, 31, 32]. In support of this, the results of this study indicate that simple measurement of total cfDNA levels is a good predictor of response, overall survival and progression-free survival in patients with metastatic breast cancer suggesting potential clinical application of a cheap and simple blood-based monitoring test.

## Conclusion

The results of this study indicate that in patients with MBC, both the amount of total cfDNA and the number of EpCAM^+^ CTCs in patient blood during the treatment period, are reflective of disease response and are indicators of overall survival. Importantly, cfDNA levels are the best predictor of disease response and PFS; however, analysis of both cfDNA and CTC counts as a paired test, provides additional prognostic information and allows further stratification of patients. In conclusion, the results of this study indicate that simple measurement of total cfDNA levels is a good predictor of response, overall survival and progression-free survival in patients with metastatic breast cancer suggesting potential clinical application of a cheap and simple blood-based monitoring test.

## Supplementary information

**Additional file 1:** Composite table with information of all the 194 samples included in the study. Information about the whole dataset included in the study.

**Additional file 2:** Composite table with information of all the 39 samples. Information about the small subset of the population used for preliminary analysis.

**Additional file 3:** Non-parametric correlation and linear regression analysis of the four biomarkers included in the study, as continuous variables, against tumour volume and number of metastatic sites. Mann-Whitney-Wilcoxon non parametric test and logistic regression analysis between stratified biomarkers and response categories against tumour volume and number of metastatics sites used as continuous variables, showing the p-value (multivariate analysis). Overall survival analysis of metastatic breast cancer patients, showing the p-value (univariate and multivariate analysis). Table including data regarding relationship between biomarkers, overall survival of patients and tumour bulk.

**Additional file 4:** Descriptive statistics of variables and clinical characteristics of patients included in the study, according to the type of disease response. Descriptive statistics of the whole population according to clinical parameters.

**Additional file 5:** Linear regression analysis between non stratified biomarkers, showing the p-value. Table containing information regarding the relationship between biomarkers as continuous variables.

**Additional file 6:** Logistic regression analysis between stratified biomarkers and normalized continuous biomarkers, showing the p-value, Odd ratio and the 95% confidence interval for this Odd ratio. Univariate and multivariate analysis. Table containing information regarding the relationship between biomarkers as categorical and continuous variables.

**Additional file 7:** Logistic regression analysis between stratified biomarkers, showing the p-value, Odd ratio and the 95% confidence interval for this Odd ratio. Univariate and multivariate analysis. Table containing information regarding the relationship between biomarkers as categorical variables.

**Additional file 8:** Logistic regression analysis between stratified clinical, response and treatment parameters and stratified biomarkers, showing the p-value, Odd ratio and the 95% confidence interval for this Odd ratio. Univariate and multivariate analysis. Table containing information regarding the interactions between biomarkers, as categorical variables, and different clinical parameters and treatment received by the patient.

**Additional file 9:** Logistic regression analysis between stratified clinical, response and treatment parameters and normalized continuous biomarkers, showing the p-value, Odd ratio and the 95% confidence interval for this Odd ratio. Univariate and multivariate analysis. Table containing information regarding the interactions between biomarkers, as continuous variables, and different clinical parameters and treatment received by the patient.

**Additional file 10:** Overall survival analysis of metastatic breast cancer patients, showing the p-value, Hazard ratio and the 95% confidence interval for this Hazard ratio. Comparison of joint analysis of new blood biomarkers (CTCs and cfDNA) against traditional biomarkers (CA15-3 and AP). Stratified biomarkers as variables. Overall survival population results from joint analysis of biomarkers.

## Data Availability

All data generated or analysed during this study are included in this published article [and its supplementary information files].
